# High response speed microfluidic ice valves with enhanced thermal conductivity and a movable refrigeration source

**DOI:** 10.1038/srep40570

**Published:** 2017-01-13

**Authors:** Chaorun Si, Songtao Hu, Xiaobao Cao, Weichao Wu

**Affiliations:** 1 School of Mechatronical Engineering, Beijing Institute of Technology, Beijing 100081, China; 2 Institute of Chemical and Bioengineering, Department of Chemistry and Applied Biosciences, ETH Zürich, 8093 Zürich, Switzerland; 3 Department of Mechanical Engineering, Tsinghua University, Beijing 100062, China

## Abstract

Due to their ease of fabrication, facile use and low cost, ice valves have great potential for use in microfluidic platforms. For this to be possible, a rapid response speed is key and hence there is still much scope for improvement in current ice valve technology. Therefore, in this study, an ice valve with enhanced thermal conductivity and a movable refrigeration source has been developed. An embedded aluminium cylinder is used to dramatically enhance the heat conduction performance of the microfluidic platform and a movable thermoelectric unit eliminates the thermal inertia, resulting in a faster cooling process. The proposed ice valve achieves very short closing times (0.37 s at 10 μL/min) and also operates at high flow rates (1150 μL/min). Furthermore, the response time of the valve decreased by a factor of 8 when compared to current state of the art technology.

Microfluidic technologies are widely used in chemical and biological analysis because they offer high analytical throughput, low sample consumption and have high integration capabilities^1^,^2^,^3^. As a key component in flow control, valves have been intensively studied, and many different types have been developed, such as pneumatic valves^4^, phase-change valves^5^ and burst valves^6^. Ice valves are of particular interest since they do not require specific on-device architectures and do not leave residues that may influence on-device analytics^7^,^8^.

Ice valves switch flow by controlling the state of the fluid phase. To stop flow, a section of the fluid in the channel is cooled to below its freezing point, and it hence solidifies, which causes the channel to be blocked. To reopen the valve, the ice is allowed to thaw by stopping the cooling process. Ice valves were first reported in 1995^9^, where a mixture of liquid and gaseous carbon dioxide was used to freeze the fluid medium. A few years later, this concept was expanded to use liquid nitrogen as the cooling medium^10^. However, such low temperature liquids and gases are hard to integrate into small devices and also have inherent safety problems^11^. In 2001, thermoelectric (TE) units were employed to freeze and thaw flow in capillaries^12^. This method was then integrated on-device, but the valve closing time was still very long (18 seconds)^13^. A more recent study used a single-level (containing only one TE unit) ice valve with a more efficient design and the valve closing time was reduced to 4.1 seconds^14^. The use of a two-level TE unit further decreased the valve closing time to 2.72 seconds^11^ by cooling the device to near 0 °C with one TE unit, and then lowering the temperature futher with the other TE unit. However, there is still a demand for improving the response speed and integration density of ice valves, especially for microfluidic applications where many processes occur in short timescales^11^.

To solve these problems, in this study an aluminium cylinder was embedded under the channel in the microfluidic platform, and a movable refrigeration source was designed to increase the response speed. The experimental results showed that the response speed was increased by a factor of 8 when compared with current state of the art ice valves (the response time is compared at the same flow rates), as well as the maximum flow rate by a factor of 10.

## Experimental Section

**Figure 1:**
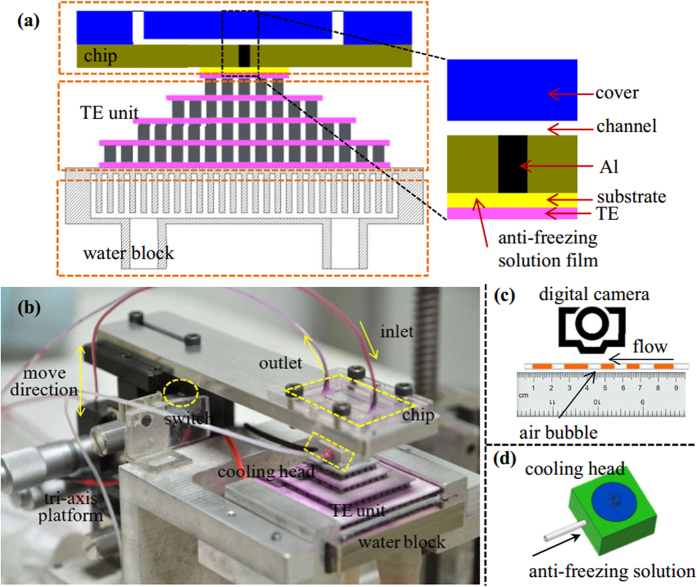
The microfluidic platform with the enhanced ice valve structure. (**a**) Schematic of the microfluidic platform, where the PMMA microfluidic device is shown in blue with the (black) aluminium cylinder embedded into the bottom channel. The TE unit is shown below the microfluidic platform, and it contacts the aluminium cylinder through a layer of anti-freeze solution (yellow layer). A water bath is used to cool the TE unit. (**b**) Photograph of the microfluidic platform in use, with each component labelled in yellow. The double-headed arrow shows the direction of motion of the tri-axial platform that allows the microfluidic device to be brought into contact with the TE unit. (**c**) Experimental set-up that allows the measurement of the valve opening and closing times using a ruler to measure the movement of the air bubbles in the tubing and a digital camera to record images. (**d**) 3-dimensional schematic of the cooling head used to dose anti-freeze onto the top of the TE unit to prevent the formation of ice.

In brief, the platform consists of a PMMA (poly(methyl methacrylate)) microfluidic device embedded with an aluminium cylinder covered with a layer of anti-freeze solution, a movable TE unit and a water cooling system (Fig. 1). When a positive electric voltage is applied to the TE unit, the top of the unit (which is closest to the microfluidic device) reaches −42.5 °C. Once the TE unit comes into contact with the aluminium cylinder in the microfluidic device, the cooling process begins and the temperature inside the fluid-filled channel is decreased until the channel is blocked by the formed ice. In order to achieve a greater temperature difference and be able to share the refrigeration source between different valves on the device, the TE unit is movable relative to the device.

A layer of anti-freeze solution, consisting of 45% ethylene glycol and 55% water (with a freezing point below −45 °C), is inserted between the TE unit and the device to reduce the contact thermal resistance. Without the anti-freeze solution, the TE unit will condense moisture from the air to form an ice layer on the top of the cooling head, which will reduce the heat transfer.

Figure 1 (a) and (b) depict the experimental apparatus used to characterize the ice valve. The microfluidic device is fixed on a mobile tri-axial platform used for adjusting the position of the device relative to the cooling head. A syringe pump (70–3408, Harvard Apparatus, USA) was used to drive the liquid flow at a constant rate into the device. The four stage TE unit (TEC4-24606, Zhong Tian, China) was mounted on top of a water block, to allow cooling of the TE unit.

The characterization of the valve was performed by measuring its closing time as a function of flow rate. To characterize the ice valve, a channel with a rectangular cross section of 300 μm × 200 μm (width × height) was fabricated in a PMMA substrate using a tri-axis CNC machine (Beijing Jingdiao Co. Ltd., China). The aluminium cylinders were made of alloy 7050-T7451 with both a height and a diameter of 1 mm. To measure the closing time of the valve, the Teflon tubing (0.3 mm interior diameter, 0.6 mm exterior diameter) at the outlet of the device was taped to a ruler. The closing time of the ice valve is defined from the point when the device contacts with the TE unit to the point that the valve closes (the flow stops). These experiments were conducted at room temperature (19.6 °C) and the cooling head was cooled to −45.2 °C (as measured using a k-type thermocouple) prior to contact with the device. The voltage applied to the TE unit remained at 12 V and the current was 5.2 A.

To keep the anti-freeze solution in a liquid state, a custom made hollow aluminium cooling head (Fig. 1(d)) was set above the TE unit to replenish the solution consumed during the experiment. Before the device came into contact with the cooling head, a small amount of anti-freeze solution was injected from the side to melt the ice on the top of the cooling head and ensure good heat conduction between the TE unit and the device.

To measure the closing time of the valve, multiple air bubbles were manually generated in the tubing connected to the outlet of the device (Fig. 1(c)), and the movement of these air bubbles was continuously recorded using a digital camera (Nikon D7000, Japan). When the device approached the cooling head, a micro switch was triggered to start a stroboscope (Drelloscop 255, Drello, Germany) to generate a bright frame in the video as a start point reference for the measurement. When no more air bubbles were visible in the tubing, the valve was deemed closed.

## Results and Discussion

**Figure 2:**
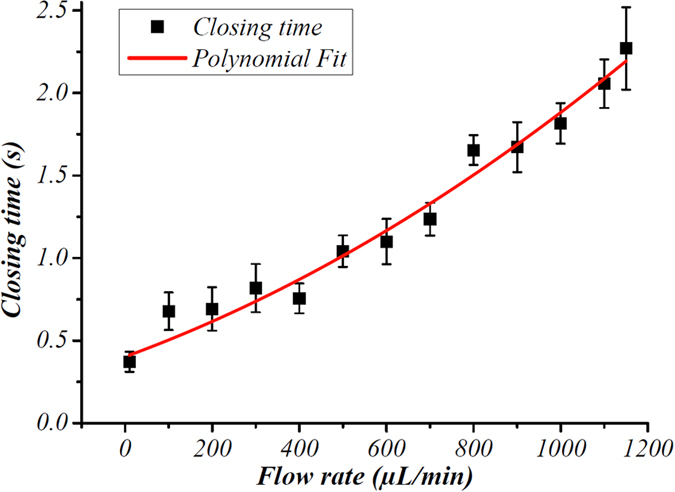
Graph showing the performance (closing time, in seconds) of the ice valve at different flow rates (μL/min). The closing time of the ice valve was measured at flow rates ranging between 0 and 1150 μL/min. The experimental data is shown in black, the red line is to guide the eye to the data trend.

Figure 2 shows the relationship between the closing time of the ice valve and the flow rate of the fluid within the microfluidic device. The closing time increases with the flow rate and is less than a second when the flow rate is below 500 μL/min. The lowest closing time for the valve is 0.37 s at a flow rate of 10 μL/min. Compared with ice valves reported in previous studies^11^,^13^,^14^, the closing time of this ice valve is over 8 times shorter at the same flow rates. Another significant improvement over previous studies is that this ice valve also exhibits good performance at high flow rates. The highest flow rate measured previously is 100 μL/min^11^,^14^, with a closing time of over 10 s. However, our ice valve still functions efficiently at a flow rate of 1150 μL/min with a closing time of only 2.5 s (Fig. 2).

We built a mathematical model to allow the prediction of the cooling time, at the same flow rate, in two different types of ice valves to allow us to compare their efficacy. Due to super cooling and the coupling of phase change and the temperature and fluid fields, it is difficult to build an accurate model to calculate the closing time of the valve mathematically. Herein we consider the cooling rate at the bottom of the channel at the same convection coefficient, which represents the ability of the system to take the heat away from the liquid in the channel. The cooling rate is determined by three main factors: the initial temperature, the heat conduction performance and the temperature difference between the cooled bottom of the channel and the flowing bulk liquid.

The initial temperature of the system determines the energy released from the liquid during the valve closing operation. In previously published work, pre-cooling the device^14^ and two-level cooling systems^11^ were used to achieve a lower initial temperature. During the cooling process, two types of energy are released from the liquid: sensible heat and latent heat. Pre-cooling will significantly decrease the sensible heat, while not affecting the latent heat and hence two-level cooling systems can be classified as another type of pre-cooling. Once the liquid in the channel starts to release its latent heat, the freezing process starts. Therefore, the pre-cooling temperature should be higher than the minimum super-cooling temperature. However, the latent heat release constitutes a large proportion of the total energy released during the freezing process. In the case of water, for example, its specific heat capacity and latent heat are *C*_water_ = 4.2 × 10^3^ J·kg^−1^·K^−1^ and *L*_water_ = 3.35 × 10^5^ J·kg^−1^. When water is cooled from room temperature (*T*_room_) to pre-cooling temperature (*T*_pre_), the energy released per unit mass, *E*_1_ in J·kg^−1^, is defined as:


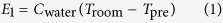


During the crystallisation stage, the energy released per unit mass, *E*_2_ in J·kg^−1^, is:


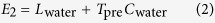


Assuming that the super-cooling temperature (*T*_super_) is −17 °C, the maximum energy released by pre-cooling is *E*_1_ = 1.55 × 10^5^ J·kg^−1^, while the total energy released during the entire cooling process is *E*_1_ + * E*_2_ = 4.9 × 10^5^ J·kg^−1^. As mentioned above, *E*_2_ (which includes the latent heat) accounts for a large proportion of the released energy, and it cannot be released by pre-cooling. Therefore, transferring the energy released during crystallization in the shortest amount of time possible is key to increasing the response speed of the ice valve. Practically, this means that better heat conduction and a higher temperature difference are important design considerations.

Higher heat conduction between the TE unit and the channel will cause a greater heat transfer rate, which is determined by the thermal conductivity and size of the material between the TE unit and the channel. The thermal conductivities of materials commonly used in microfluidic platforms are listed in Table 1. It is obvious that the thermal conductivity of the aluminium alloy is much larger than that of other materials, and it was therefore used in this study to improve the performance of the ice valve.

The heat transfer coefficient, *k*_1_, of our valve is^15^:


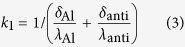


where *δ*_Al_ is the thickness of the aluminium alloy and has a value of 1 mm, *δ*_anti_ is the thickness of the anti-freeze layer and has a value of 0.023 mm, and *λ*_Al_ and *λ*_anti_ are the thermal conductivity of the alloy and of the anti-freeze solution respectively. The value for *k*_1_ obtained using Equation 3 is 1.28 × 10^4^ W·m^−2^·K^−1^.

In traditional ice valves, the thermal conductive layer normally consists of a substrate, such as glass, and thermal silicone grease is used to improve the thermal conductivity. In such a case, the heat transfer coefficient, *k*_2_, is^15^:


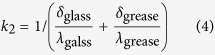


where *δ*_glass_ is the thickness of the glass and has a value of 1 mm, *δ*_grease_ is the thickness of the silicone grease, and *λ*_glass_ and *λ*_grease_ are the thermal conductivities of glass and silicone grease respectively. Since *δ*_grease_/*λ*_grease_ is so much smaller than *δ*_glass_/*λ*_glass_, it can be ignored and hence the value obtained for the heat transfer coefficient, *k*_2_, of the traditional ice valve using Equation 4 is 6.88 × 10^2^ W·m^−2^·K^−1^. Since *k*_1_ is much larger than *k*_2_, the heat conduction performance of the ice valve proposed in this study is much better than that of traditional ice valves.

In addition to the heat conduction performance and to the initial temperature, the temperature of the cold side of the TE unit is also an important factor that influences the response speed of the ice valve. A lower temperature will speed up the cooling process by generating a greater temperature difference between the TE unit and the channel. It is worth noting that although increasing the power of the TE unit will make the temperature on the cold side of the TE unit lower, in traditional ice valves this takes a significant amount of time due to the thermal inertia of the TE unit^14^. In this study the TE unit was designed to be movable and hence the device and the cooling head are separate. Thus, the cooling head can be kept at a much lower temperature at all times prior and during contact with the device and hence the thermal inertia in the system is removed. Once the device is in contact with the cooling head, the bottom of the device is effectively in contact with a lower temperature and the cooling process is surely accelerated.

The factors that influence the performance of the ice valve were investigated using a 2D simulation model solved in COMSOL Multiphysics Modeling Software (version 5.2) for both traditional ice valves and the ice valve proposed in this study. The response speed of the ice valves is determined by the heat conductivity of the substrate in contact with the TE unit and the temperature of the cold side of the TE unit. To highlight the performance enhancements achieved by our ice valve design, the traditional ice valve is simulated in a glass microfluidic device, which should theoretically show a better performance than a PMMA microfluidic device with the same structure. The traditional ice valve has a non-movable TE unit integrated into the device and the thermal contact resistance can therefore be ignored. For both platfroms, the length and height of the channel are 20 mm and 0.2 mm, respectively, and the widths of the TE unit and the aluminium cylinger for our platform are 6 mm and 1 mm respectively. The governing equation for the simulation model is the heat equation for conductive and convective heat transfer:


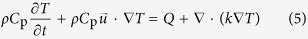


where *C*_p_ is the specific heat capacity (J·kg^−1^·K^−1^), *T* is the temperature (K), *k* is the thermal conductivity (W·m^−1^·K^−1^), *ρ* is the density (kg·m^−3^), *u* is the velocity vector (m·s^−1^), and *Q* is a sink or source term (which is set to zero because heat is neither produced nor consumed in the device).

In the channels, the velocity field of the fluid flow is defined by an analytical expression that approximates a fully developed laminar flow. The velocity components in the *y* direction are set to zero. The expression:


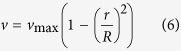


gives the *x* component of the velocity, where *v*_max_ is the maximum velocity (ms^−1^) that arises in the middle of the channel, *r* is a variable defined as the distance from the center of the channel to the measurement point, and *R* is equal to half the channel height such that *r*_max_ = *R*. The simulation accounts for heat conduction in the device, as well as convection in the aluminium cylinder and in the anti-freeze solution. This COMSOL model allows us to examine the effects of various factors on valve performance. The COMSOL model was defined as a fully insulated system, except for the inlet and outlet boundaries, and the boundary that comes in contact with the TE unit in the channel. At the inlet, a constant temperature was specified as 20 °C (room temperature). At the boundary that comes in contact with the TE unit, a constant temperature was specified at −45 °C since that is the approximate temperature at the top of the cooling head. At the outlet, convection dominates the transport of heat, and thus, the convective flux boundary condition, 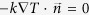, was applied.

**Figure 3:**
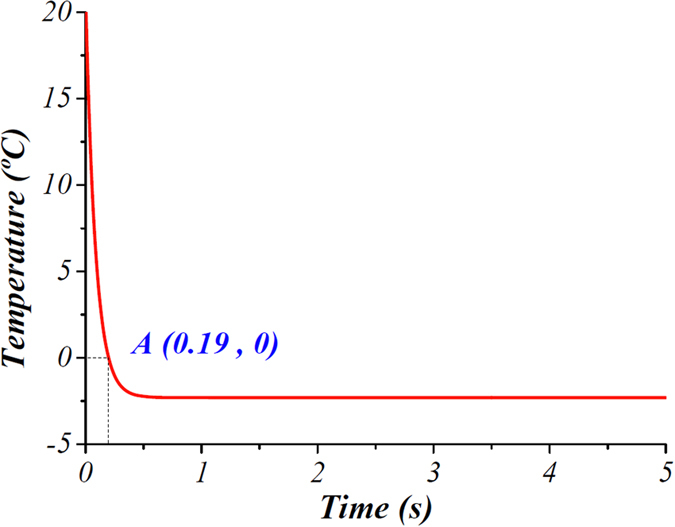
Temerature at the bottom of the channel, predicted using a COMSOL model, along the time at a flow rate of 96 μL/min. At point A, the temperature reaches 0 °C, the time at which is defined as cooling time.

Figure 3 shows the temperature at the bottom of the channel, predicted using a COMSOL model, at flow rate of 96 μL/min for traditional ice valve (glass thickness of 0.5 mm). As the temperature difference between the flow channel and the cooling head is huge at the beginning, the temperature decreases dramatically. At point A, the bottom of the channel reaches 0 °C, where the time is defined as cooling time.

**Figure 4:**
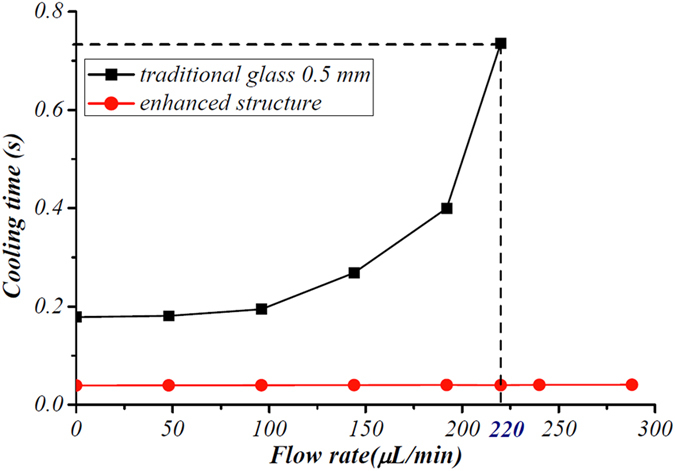
Cooling time (in seconds), predicted using a COMSOL model, required for the ice valve to reach 0 °C at different flow rates for both a traditional ice valve (data in black, glass thickness of 0.5 mm) and the ice valve with an enhanced thermal conductivity structure developed in this study (data in red). The traditional ice valve shows a significant increase of cooling time against the flow rate. While cooling time of the valve with an enhanced thermal conductivity keeps much lower. This shows the higher cooling speed of our valve than that of traditional ice valves.

Figure 4 shows the time predicted by our COMSOL model required to decrease the temperature to 0 °C in the traditional ice valve and in our ice valve with the enhanced thermal conductivity structure. The temperature at the bottom of the flow channel is calculated with the time at a defined flow rate. At short timescales, the cooling speed is high. Cooling time increases sharply at a flow rate of 200 μL/min. The highest time taken to reach 0 °C is predicted to be at a flow rate of 220 μL/min, and the temperature will never reach 0 °C when the flow rate is above 240 μL/min. The temperature of the fluid within the channel dramatically decreases to 0 °C within a short period (0.039 s to 0.044 s) at flow rates ranging from 0 to 1200 μL/min due to the integration of the Al cylinder (data for higher flow rates for our ice valve are now shown in Fig. 4. Thus, our ice valve with the enhanced thermal conductivity structure exhibits a higher response speed, even when compared to a traditional ice valve fabricated in a more effective material.

**Figure 5:**
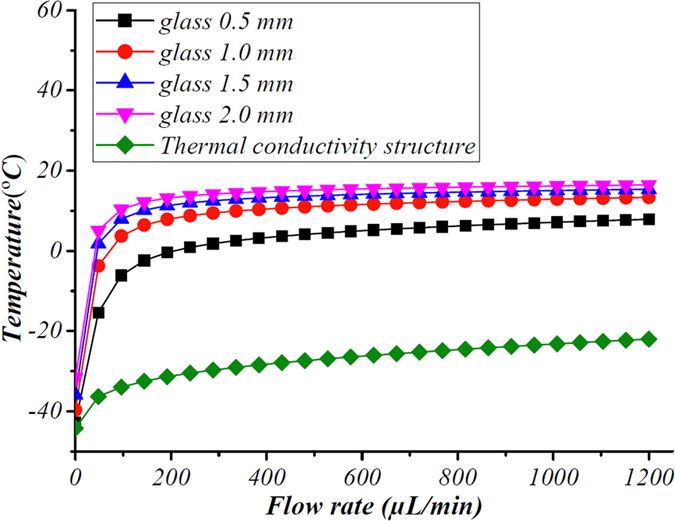
Graph showing the equilibrium temperature reached by the fluid within the microfluidic channel at a range of flow rates, as predicted by our COMSOL model. In each case the temperature is measured after 30 seconds. Data are shown for both a traditional ice valve in a glass microfluidic device with different glass thicknesses (with different thickness: 0.5 mm, 1 mm, 1.5 mm, 2 mm), and for our ice valve with the enhanced thermal conductivity structre. The temperature increases with flow rate and the thichness of the chip. When the flow rate increases to certain flow rate, the temperature reachs to higher than 0 °C, which means it’s impossible for the valve to close. But in our valves, the temperature could still keep lower than 0 °C. Thus our valve has a much wider work range.

At any given flow rate, and given sufficient time, the COMSOL model shows that the flow within the channel reaches a constant temperature. The equilibrium temperature at each flow rate is shown in Fig. 5 with both the traditional and our ice valve. In each case the temperature was measured after 30 seconds, however, when the flow rate is 0 μL/min, the temperature was still decreasing at the 30 s timepoint, so the equilibrium temperature may be lower. The data in Fig. 5 shows that the equilibrium temperature increases with the flow rate because more heat is taken into the channel by the flow at a higher flow rate. When the flow rate reaches a certain value, the equilibrium temperature will never reach 0 °C, thus indicating that the flow will never freeze. However, the ice valve with the enhanced thermal conductivity structure proposed in this study can obtain a significantly lower temperature (below −20 °C) even at high flow rates of 1200 μL/min. The COMSOL simulation shows that the ice valve with the enhanced thermal conductivity structure exhibits better performance at a high flow rates than traditional ice valves, which is confirmed by its efficient performance at high flow rates during the experiment.

The design of this platform such that the TE unit is a separate module from the microfluidic device increases the integration density of the ice valves. In traditional ice valves, the TE units are fixed to the device and therefore the size of the TE unit limits the integration density. In our platform, the cold area is concentrated solely in the zone contacting the aluminium cylinder and is therefore not constrained by the TE unit itself. Thus, decreases in the size of the Al cylinder will further increase the integration density. Finally, the enhanced performance exhibited by our ice valve is not dependant on the material from which the microfluidic device is fabricated since the thermal conductivity of the Al alloy is over 100 times higher than that of most commonly used materials for device fabrication (Table 1).

## Conclusions

Herein we show a simple and robust ice valve with enhanced thermal conductivity and a movable refrigeration source. This ice valve is capable of very short closing times (0.37 s) when compared to current literature and is able to operate at extremely high flow rates (1150 μL/min). We provide mathematical and COMSOL models to describe the design and behaviour of our ice valve compared to traditional ice valves. The response speed of our valve was over than 8 times faster than current ice valves operating at similar flow rates and it has a series of other advantages such as improved integration density of the valves on device and no limitation with regards to type of device material required.

## Additional Information

**How to cite this article**: Si, C. *et al*. High response speed microfluidic ice valves with enhanced thermal conductivity and a movable refrigeration source. *Sci. Rep.* **7**, 40570; doi: 10.1038/srep40570 (2017).

**Publisher's note:** Springer Nature remains neutral with regard to jurisdictional claims in published maps and institutional affiliations.

## Figures and Tables

**Table 1:** Thermal conductivity of materials commonly used in the fabrication of microfluidic devices.

	glass	PMMA	PC	air	water	Al alloy	anti-freeze
thermal conductivity (Wm^−1^K^−1^)	1.1	0.18	0.2	0.023	0.5	157	0.32

PC is polycarbonate and the aluminiun alloy is 7050-T7451. Data from ref. 16.
